# The Institutional Worker

**Published:** 1911-10-14

**Authors:** 


### The Institutional Worker

**Being a Special Supplement to "The Hospital"**

### CONGRATULATORY MESSAGES FROM OUR READERS.

We have received several commendatory letters in praise of The Hospital, and have pleasure in giving publicity to a few out of the numerous communications which have reached our offices.

Dear Sir,—Your new and enlarged and very much more interesting "Hospital" will be largely read by philanthropic workers of all kinds, lay and official.

Editor, The Hospital.

Dear Sir,—I sincerely hope that this is the dawn of our principal organ. It is delivered through my newsagent to me regularly, and I feel sure I can get it more widely circulated in this district. Wishing you every success.

The Hospital, London, W.C.

Royal Infirmary, Sunderland.

Dear Sir,—As a reader of The Hospital since its first issue I offer my congratulations on the distinct improvement in the journal.—Yours faithfully, Thomas Robinson, Secretary.

Editor, The Hospital,
Southampton Street, Strand, W.C.

The Hospital has just entered upon its twenty-sixth year. In order to extend its usefulness as a medium of enlightenment on such important subjects as health, administrative medicine, and national insurance, the management have decided to make it for the future a penny weekly newspaper.—*Sunday Times.*

The Hospital enters upon its twenty-sixth year with the current number. The price is reduced to one penny, and it becomes a weekly newspaper of administrative medicine and institutional life, administration, national insurance, and health. Sir Henry C. Burdett says :—"The momentous changes which the establishment of national insurance must cause will, I hope, result, if we are wise and prudent, by co-operation and intercommunication, in one great organised administration, which will remove existing abuses, prevent overlapping, and secure and provide the maximum of facility for all classes to obtain exactly the hospital, medical, and other care which the individual may require when overtaken by accident or disease."—*Belfast News Letter.*

### GENERAL REQUIREMENTS.

There are very few people who are not wanting some article for which they feel they cannot afford to pay the full price, but who would gladly purchase what they require if they could obtain it at a reduced cost. The columns of The Hospital are open for Wanted and For Sale announcements of every description. Those of our readers who wish to dispose of articles for which they have no further use are invited to send their advertisements to The Hospital. The charge is 6d. for 14 words and 3d. for every 6 additional words. Each advertisement is numbered, so that no name or address is published unless desired, and therefore all transactions are strictly private.

### RECOMMEND "THE HOSPITAL" TO YOUR FRIENDS.

The first number of the new Hospital has been well received throughout the country, and there is abundant testimony that the Journal is highly appreciated for the excellence of its contents. 25,000 copies were circulated, and the value of The Hospital to advertisers is fully recognised. It is the only Journal appealing to the institutional worker of all grades.

### INSTITUTIONAL WORKERS:

Are you seeking an appointment? A large number of vacancies are announced in this week's issue of The Hospital. The newspaper is on sale at all newsagents and bookstalls throughout the country.

The Hospital has for upwards of a quarter of a century held an important and unique position as the trusted exponent of Medical and Institutional life and work. It is now published at the popular price of one penny.

### LEST YOU FORGET.

Send your advertisements for The Hospital to reach us not later than Wednesday morning.

The departments of the paper will include :—The Hospital and Institutional World; The Week's Work; State and Administrative Medicine; National Insurance; Institutional Economics; Co-operation and Inter-communication; National Health; The Care of the Individual; School Clinics; Delay and Comforts of Old Age; Maternity and Mothering; The Housekeeper in the Institution; The Making of the Modern Practitioner; Feeble-minded and Defective Children; Practical Aids for Institutional Workers; Friendly Societies and their Work; General and Special Hospitals; Asylums, Infirmaries, and Homes; Bye-ways of Medical and Nursing Care; Sick Cookery and Menus; Sanatoria, their Use and Abuse; Electricity in Treatment; Architecture, Engineering, and Construction; Physic in the Making; The Question Box for Practical Information; Off Duty Hours; Exercise and Locomotion.

### INTERESTING ARTICLES.

The Editor of The Hospital invites from Institutional workers contributions based upon their experience in connection with administration, finance, construction, or any practical subjects. Photographs illustrating an article will also be welcome.

No article should exceed 1,100 words, and, if accepted, one guinea for a contribution of this length will be paid to the writer after publication. Each communication should be accompanied by a stamped directed envelope for the return of the MS. if found unsuitable.

Contributions should be written, or preferably typed, on one side of the paper only, and all articles sent in are accepted upon the distinct understanding that they are forwarded to The Hospital only.

**Suggestions Invited.**

The Editor will welcome suggestions for the establishment of any new section in The Hospital, and will be glad, on a written request, to supply information on any subject of interest or importance to institutional workers in any part of the world.

LISKEARD UNION.

### APPOINTMENTS VACANT.

### Poor-Law Vacancies.

### Appointments Vacant.

NURSE (£30), Ellesmere Union. Mr. W. G. Thomas, C. to G., Ellesmere. Oct. 14.

COOK AND ASSISTANT MATRON (£25), Forehoe Guardians. Mr. W. P. Smith, C. to G., Wymondham. Oct. 14.

PROBATIONER (£14), Worcester Union. Mr. J. G. Sheild, C. to G., Worcester. Oct. 16.

FOSTER-PARENTS (married couple) (£30 and £20), Blean Union. Mr. J. E. Burch, C. to G., 39 Castle Street, Canterbury. Oct. 16.

NURSE (£26), Hastings Union. Mr. A. R. Inskipp, C. to G., Hastings. Oct. 16.

RELIEF FOSTER-MOTHERS (2) (£30 each), Camberwell Guardians. Mr. C. S. Stevens, C. to G., Peckham Road, S.E. Oct. 16.

ASSISTANT NURSE (£20), Cricklade and Wootton Bassett Union. Mr. H. Bevir, C. to G., Wootton Bassett Oct. 16.

ASSISTANT NURSE (£20), Monmouth Union. Mr. J. Smith, C. to G., Monmouth. Oct. 17.

FOSTER-MOTHER (£25), Wellingborough Union. Mr. T. J. Morgan, C. to G., Wellingborough. Oct. 17.

NURSE (£30), Fylde Union. Mr. F. H. Brown, C. to G., Wesham, Kirkham. Oct. 17.

WARD NURSE (£20), Southwark Union. Mr. S. Wood, C. to G., Ufford Street, Blackfriars, S.E. Oct. 17.

MALE INFIRM ATTENDANT (£30), Southwark Union. Mr. S. Wood, C. to G., Ufford Street, Blackfriars, S.E. Oct. 17.

ASSISTANT NURSE (£25), Exeter Guardians. Mr. A. Snell, C. to G., Exeter. Oct. 18.

ASSISTANT NURSE (£25), Christchurch Union. Mr. A. Druitt, C. to G., Christchurch. Oct. 18.

LABOUR MASTER (£25), Farnham Union. Mr. E. Crundwell, C. to G., Farnham. Oct. 18.

ASSISTANT MALE ATTENDANT (£35), Ecclesall Bierlow Union. Mr. J. E. Moulding, C. to G., The Edge, Sheffield. Oct. 18.

MALE ATTENDANT (£25), Exeter Guardians. Mr. A. Snell, C. to G., Exeter. Oct. 18.

MIDWIFE (£36), Paddington Guardians. Mr. H. F. Aveling, C. to G., 313-319 Harrow Road, W. Oct. 18.

ASSISTANT NURSE (£25), Liskeard Union. Mr. A. de Castro Glubb, C. to G., Liskeard. Oct. 18.

ATTENDANTS ON MALE IMBECILES (2) (£27 10s.), Preston Union. Mr. J. Clarke, C. to G., Preston. Oct. 19.

ASSISTANT NURSE (£22), Congleton Union. Mr. H. Ferrand, C. to G., Sandback. Oct. 20.

FEMALE GENERAL ASSISTANT (£18), Pockington Union. Mr. T. Robson, C. to G., Pocklington. Oct. 20.

ASSISTANT MASTER AND BOOKKEEPER (£80), South Manchester Guardians. Mr. D. S. Bloomfield, C. to G., All Saints, Manchester, Oct. 20.

Master and Matron (£90 and £60), Preston Union. Mr. J. Clarke, C. to G., Preston. Oct. 20.

Nurse (£25), Birmingham Guardians. Mr. C. Fletcher, C. to G., Birmingham. Oct. 20

Assistant Nurses (£30), Llanelly Union. Mr. D. C. Edwards, C. to G., Llanelly. Oct. 23.

Sister (£30), Camberwell Guardians. Mr. C. S. Stevens, C. to G., Peckham Road, S.E. Oct. 20.

Superintendent Nurse (38), Malling Union. Mr. F. J. Allinson, C. to G., West Malling. Oct. 23.

Assistant Nurse (£22), Malling Union. Mr. F. J. Allinson, C. to G., West Malling. Oct. 23.

Assistant Matron (£40), Southampton Union. Mr. A. J. Walden, C. to G., Southampton. Oct. 23.

Nurse (£35), Freebridge Lynn Union. Mr. W. Cross, C. to G., King's Lynn. Oct. 26.

Storekeeper and Assistant Clerk (£40), Merthyr Tydfil Union. Mr. F. T. James, C. to G., Merthyr Tydfil. Oct. 26.

Nurses (2) (£30 each), Winchester Guardians. Mr. F. Faithfull, C. to G., Winchester. Oct. 30.

Valuer, Blean Union. Mr. J. E. Burch, C. to G., 39 Castle Street, Canterbury. Oct. 30.

Master's Second Clerk (£30), Islington Guardians. Master of the Workhouse, St. John's Road, Upper Holloway, N.

Nurse (£36), Ipswich Guardians. Mr. L. W. Greenhalgh, C. to G., Ipswich.

Infants' Attendant (£22), Bicester Union. Mr. W. Parks, Master of the Workhouse, Bicester.

Probationers (£10), Rochdale Union. Mr. R. A. Leach, C. to G., Rochdale.

Male Relief Officer (30s. weekly), Brentford Union. Mr. W. Stephens, C. to G., Isleworth, W.

Storekeeper and Assistant Clerk (£40), Merthyr Tydfil Union. Mr. F. T. James, C. to G., Merthyr Tydfil. Oct. 26.

Children's Attendant (£20), Lexden and Winstree Union. Matron of the Workhouse, Stanway, Colchester.

Nurse (£27), Wilton Union. Mr. G. M. Wilson, C. to G., Wilton, Salisbury.

### Situations Vacant.

Barber and Bath Attendant. (£28), Hammersmith Guardians. The C. to G., Goldhawk Road, Shepherd's Bush. Oct. 16.

Cook (£25), Bosmere and Claydon Union. Mr. R. M. Cook, C. to G., 6 Providence Street, Ipswich. Oct. 16.

Head Laundress (£35), Greenwich Union. Mr. S. Saw, C. to G., East Greenwich. Oct. 16.

Porter and Portress (married couple) (£30 and £25), Barnsley Union. Mr. C. J. Tyas, C. to G., Barnsley. Oct. 16.

Barber (£30), Wolverhampton Union. Mr. F. Harrison, C. to G., Wolverhampton. Oct. 16.

Assistant Laundress (£20), Holborn Union. Matron of the Workhouse, Mitcham, Surrey. Oct. 16.

Female Cook (£30), Oxford Guardians. Master of the Workhouse, Oxford. Oct. 17.

Female Cook (£30), Brighton Guardians. Mr. B. Burfield, C. to G., Brighton. Oct. 18.

Assistant Needlemistress (£20), Brighton Guardians. Mr. B. Burfield, C. to G., Brighton. Oct. 18.

Laundress (£25), Bosmere and Claydon Union. Matron of the Workhouse, Barham, near Ipswich.

Female Assistant Cook (£25), Kensington Guardians. Matron of the Infirmary, Marloes Road, Kensington.

Porter (£25), Skipton Union. Mr. M. R. Knowles, C. to G., Skipton.

Laundrymaid (£20), St. Marylebone Guardians. Matron of the Infirmary, Notting Hill, W.

Needlemistress (25), Bethnal Green Guardians. Matron of the Workhouse, Waterloo Road, Victoria Park, N.E.

Barber and Bath Attendant (£25), Isle of Thanet Union. Mr. C. Taylor, C. to G., Minster, near Ramsgate.

### Municipal Vacancies.

### Appointments Vacant.

Clerk to Humber Conservancy Board. Mr. A. W. Franklin, Conservancy Buildings, Hull. Oct. 14.

Charge Nurse (£35), Ealing Isolation Hospital. Mr. G. E. Brydges, Town Clerk, Ealing. Oct. 14.

Assistant Nurse (£20), Stafford Isolation Hospital. The Town Clerk, Stafford. Oct. 14.

Assistant Inspector of Weights and Measures (£100), West Ham T. C. Mr. F. E. Hilleary, Town Clerk, West Ham. Oct. 16.

Engineer for Cold-Air Stores (£3 weekly), Manchester T.C. The Town Clerk, Manchester. Oct. 16.

Surveyor and Inspector of Nuisances (£120), Llantarnam U.D.C. Mr. H. H. Haden, Clerk to the Council, Pontypool. Oct. 17.

Junior Clerk (Surveyor's) (£1 weekly), Margate T.C. Mr. E. Brooke, Town Clerk, Margate. Oct. 18.

Inspector of Nuisances (£80), Derby T.C. Dr. A. E. Brindley, Medical Officer of Health, Derby. Oct. 18.

Professional Auditor, Pwllheli T.C. Mr. E. R. Davies, Town Clerk, Pwllheli. Oct. 20.

Clerk (£200), Cornwall County Asylum. Mr. M. F. Edyvean, Clerk to the Visitors, Bodmin. Oct. 21.

Accountant Clerk (£156), Southampton C.C. Mr. W. J. Taylor, County Surveyor, Winchester. Oct. 23.

### Situations Vacant.

Town Hall Keeper (£26 and residence), Wokingham T.C. Mr. J. May, Town Clerk, Wokingham. Oct. 28.

Laundrymaid (£20), Brighton County Asylum. Medical Superintendent of the Asylum, Hayward's Heath.

Gardener (26s. weekly), Salop County Asylum. Medical Superintendent of the Asylum, Shrewsbury.

### Miscellaneous Vacancies.

### Appointments Vacant.

School Nurse (£80), Aberdare Education Committee. Mr. T. Botting, Director of Education, Aberdare. Oct. 19.

Assistant Clerk (£35 resident), Barnsley Hall Asylum. Medical Superintendent of the Asylum, Bromsgrove.

School Nurse Attendant (£60), Birmingham Education Committee. Mr. J. A. Palmer, Secretary for Education, Edmund Street, Birmingham.

Bandsmen-Attendants (£31), L.C.C. Medical Superintendent, Hanwell Asylum, W.

General Assistant for Girls' Industrial School (£30), Leeds Education Committee. Mr. J. Graham, Secretary for Education, Leeds.

### Situations Vacant.

Assistant Laundress (£20), Monyhull Colony for Epileptics. Matron of the Colony, King's Heath, Birmingham.

